# A cross-sectional study from Qatar on the effect of influenza vaccination on the severity of COVID-19

**DOI:** 10.1097/MD.0000000000035107

**Published:** 2023-09-15

**Authors:** Merlin Thomas, Shanima Ismail, Mansoor Hameed, Sabeeha Sayed Tarique Kazi, Prem Chandra, Tasleem Raza, Theresa Paul, Hisham Abdul Sattar, Aasir M. Suliman, Sara Saeed Ibrahim Mohamed, Ezzedin A. Salam Ibrahim, Eihab Abd Alla Abd Elrahim Subahi

**Affiliations:** a Department of Pulmonary Medicine, Hamad General Hospital, Doha, Qatar; b Department of Medicine, Weil Cornell Medical College, Doha, Qatar; c Medical Research Center, Academic Health Systems, Hamad Medical Corporation, Doha, Qatar; d Department of Medical Intensive care, Hamad General Hospital, Doha, Qatar; e Department of Geriatric Medicine, Hamad General hospital, Doha, Qatar; f Department of Allergy and Immunology, Hamad General hospital, Doha, Qatar; g Department of Internal Medicine, Hamad General Hospital, Doha, Qatar.

**Keywords:** COVID-19, flu vaccine, influenza VACCINE, SARS CoV-2, severity of COVID-19

## Abstract

To assess and compare the severity of corona virus disease 2019 (COVID-19) infection in patients with and without a history of influenza vaccination. In this cross-sectional study descriptive statistics were used to analyze COVID-19-related parameters, including demographics, comorbidities, and severity. Normally distributed data with mean, standard deviation, and 95% confidence interval (CI) were reported, while non-normally distributed data was presented with median and inter-quartile range. Categorical data was summarized using frequencies and percentages. Associations were assessed using Pearson Chi-square, Fisher Exact, *t* test, or Mann–Whitney *U* test. Univariate and multivariate logistic regression methods were used to evaluate the relationship between disease severity, clinical outcomes, influenza vaccination status, and other predictors. Significance was considered for p values < 0.05. Statistical analyses were done using SPSS V.27.0 (IBM Corp) and Epi Info (CDC) software. Between March 2020 and December 2020 before the availability of COVID-19 vaccination, 148,215 severe acute respiratory syndrome corona virus 2 positive patients were studied, with 3519 vaccinated against influenza, and 144,696 unvaccinated. After random sampling at 1:2 ratio, the final analysis included 3234 vaccinated and 5640 unvaccinated patients. The majority (95.4%) had mild or asymptomatic COVID-19, while 4.6% had severe or critical cases as defined by World Health Organization severity grading. Multivariate logistic regression analysis revealed that the vaccinated group had significantly less severe (adjusted odds ratio [OR] 0.683; 95% CI 0.513–0.911, *P* = .009) and critical (adjusted OR 0.345; 95% CI 0.145–0.822, *P* = .016) COVID-19 and were less likely to require oxygen therapy (adjusted OR 0.696; 95% CI 0.531–0.912, *P* = .009) after adjusting for confounders like age, gender and comorbidities. No significant differences in Intensive care unit admissions (adjusted OR 0.686; 95% CI 0.425–1.11, *P* = .122), mechanical ventilation (adjusted OR 0.631; 95% CI 0.308–1.295, *P* = .209) and mortality (adjusted OR 1.105; 95% CI 0.348–3.503, *P* = .866) were noted between the 2 groups. Influenza vaccination may significantly reduce the severity of COVID-19 but has no significant effect on intensive care unit admissions, mechanical ventilation and all- cause mortality.

## 1. Introduction

Corona Virus disease 2019 (COVID-19) has massively impacted life, health, and society globally. The novel severe acute respiratory syndrome corona virus 2 (SARS-CoV-2) infection triggered significant morbidity and mortality due to its contagious nature, relatively high fatality rate and absence of definitive therapeutic options. After its declaration as a global pandemic on 11^th^ March 2020,^[1]^ various preventive strategies were implemented, some more effective and practical than others. Influenza vaccination has been reported to be associated with a lower risk of severe COVID-19^[2–7]^ in some studies while others have found no such association.^[8]^ The influenza vaccination mediated protection against COVID -19 could be due to various hypothetical mechanisms. Structural similarities between both viruses (single-stranded Ribo nucleic acid viruses encapsulated by nucleoprotein),^[9]^ bystander immunity, and cross-reaction in immune response from interaction between influenza vaccine and SARS-CoV-2 infection are some of these mechanisms.^[10,11]^

One factor contributing to conflicting results between studies on impact of influenza vaccination on COVID-19 severity could be the varying ethnicity of the population. The population of Qatar is unique and diverse, constituting 8.6% of nationals and 91.4% of expatriates composed of people from over a hundred different nationalities, among the adult population (above 15 years) as per the 2020 December census.^[12]^ As of January 2023, Qatar reported a total of approximately 491,000 cases of COVID-19, with 685 deaths.^[13]^ Considering the foregoing, we devised this study to examine the effect of influenza vaccination on the severity of SARS-CoV-2 infection. From March 2020 to December 2020, we looked at the severity, hospitalization, and fatality rates of patients who tested positive for SARS-Cov-2 and its association with influenza vaccination status.

## 2. Methods

### 2.1. Study design and ethical approval

The study was designed as a cross-sectional analysis between period March 01, 2020 to December 31, 2020 at Hamad Medical Corporation (HMC), Qatar, the tertiary healthcare provider in the country. The study (MRC-01-20-774) was approved by Medical Research center, HMC, Doha, Qatar.

## 3. Data source

The first COVID-19 vaccination received its emergency use authorization in early December 2020 and was not available worldwide or in Qatar during the study period. All COVID-19 positive patients were treated solely at HMC. Any patient with symptoms suggestive of COVID-19, close contacts of confirmed COVID-19 cases and travelers entering the country were all tested for SARS-CoV-2. HMC Virology laboratory was the only center within the state of Qatar testing for SARS-CoV-2. Respiratory samples taken at any designated government or private testing facility of Qatar were sent to the HMC virology laboratory for reverse- transcription-polymerase chain reaction analysis. In patients who had multiple tests for SARS-CoV-2, the initial test was taken as the reference. All patients who tested positive for SARS-CoV-2 during the study period were divided into 2 groups based on documented influenza vaccination status in the previous influenza season, which ran from September 01, 2019 to March 30, 2020. Influenza vaccination data is captured in the HMC and Primary Health Care Corporation medical records after a patient receives the vaccine. Vaccinations administered in private healthcare institutions and during immunization camps are not registered in HMC records. Patients having a negative record of influenza vaccination were contacted by phone to confirm if they received the vaccine at a private healthcare facility or at an immunization camp. Those who had the influenza vaccine after testing positive for SARS-CoV-2 were excluded from the study. HMC 215 Business Analytics section conducted the search for all positive SARS-CoV-2 positive cases using HMC electronic medical record data base for the study period.

Search of electronic medical record was done with the following criteria:

### 3.1. Inclusion criteria

1. Age ≥ 18 years.
2. Positive SARS-CoV-2 RT-PCR in any respiratory secretions.

### 3.2. Exclusion criteria

1. Respiratory illnesses tested negative for SARS-COV-2 RT-PCR.

## 4. Sociodemographic and clinical variables

Baseline characteristics, clinical variables and COVID-19 outcomes were obtained from medical record reviews. Severity of COVID was classified based on the World Health organization severity grading^[14]^as follows:

Asymptomatic: Patients who tested positive for COVID-19 but lack clinical or radiological abnormalities.

Mild disease: Patients with a positive COVID-19 test and any of the following:

1. Symptoms-fever, fatigue, dry cough, anorexia, runny nose or sputum production.
2. Asymptomatic with new radiological changes (e.g., ground glass changes) suggestive of pneumonia.

Severe/Critical disease: Patients with a positive COVID-19 test and any of the following:

1. Dyspnea (respiratory rate ≥ 30 breath/minutes).
2. Hypoxia (oxygen saturation ≤ 93%).
3. Radiological changes involving ≥ 50% of the lung.
4. Severe disease complications (e.g., respiratory failure, requirement of mechanical ventilation, septic shock, or non-respiratory organ failure).

## 5. Outcomes

Primary outcome: Association between influenza vaccination status and severity of COVID-19 infection.

Secondary outcome: To compare the clinical outcomes including rate of hospitalization, intensive care admission, length of stay in intensive care, oxygen therapy, need for assisted ventilation and mortality among the influenza vaccinated and unvaccinated patients with COVID-19 infection.

## 6. Statistical analysis

Along with the variables for the primary and secondary outcomes, demographic data, comorbidities, and smoking status were also reviewed. Descriptive statistics were used to summarize and determine the sample characteristics and distribution of various considered parameters related to demographics, comorbidities, and severity of COVID-19. The normally distributed data and results were reported with mean and standard deviation with a corresponding 95% confidence interval (CI); the remaining results were reported with median and inter-quartile range. Categorical data is summarized using frequencies and percentages. Association between various demographic data, comorbidities, influenza vaccination status and severity of COVID-19 was assessed using Pearson Chi-square or Fisher Exact tests as appropriate (for categorical variables) and *t* test or Mann–Whitney *U* test (for quantitative outcome measures). Univariate and multivariate logistic regression methods were used to evaluate and quantify the association between disease severity and clinical outcomes including influenza vaccination status as main exposure variable and along with other potential predictors such as demographics and various comorbidities. All *P* values presented were 2-tailed, and *P* values < .05 were considered statistically significant. All statistical analyses were performed using statistical packages SPSS V.27.0 (IBM Corp) and Epi Info (CDC) software.

## 7. Results

**Figure 1.**
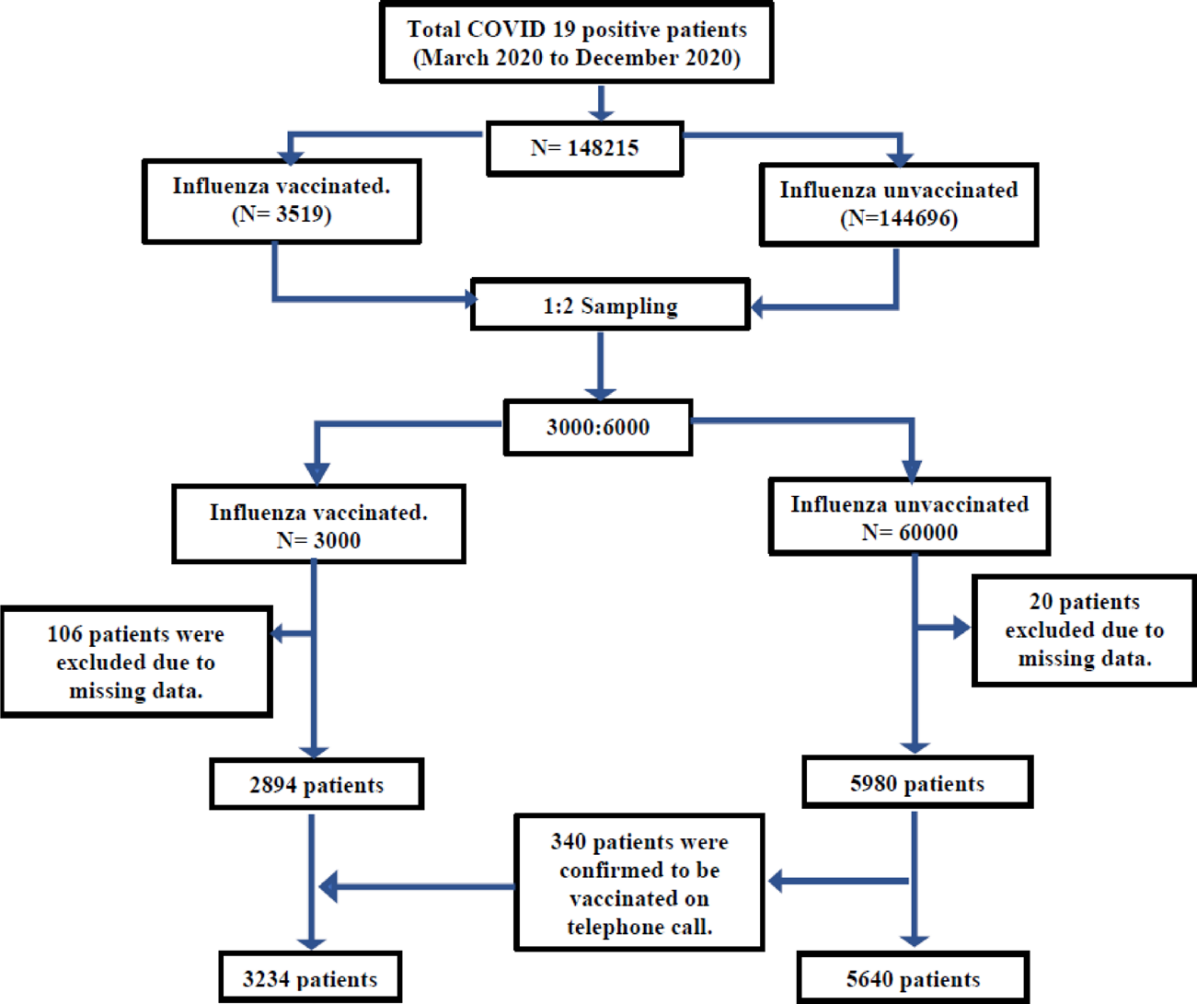
Flow chart describing participant selection for investigating the association between influenza vaccination and severity of COVID-19. COVID-19 = corona virus disease 2019.

From March 2020 to December 2020, a total of 148,215 patients with verified SARS-CoV-2 RT-PCR positive results were identified. According to medical records, 3519 individuals were vaccinated against influenza during the last influenza season (September 2019 to March 2020) while 144,696 people were unvaccinated. Random sampling was done using Microsoft excel at a ratio of 1:2 to select 3000 patients from the vaccinated group and 6000 patients from the unvaccinated group. Due to missing data, 106 and 20 patients were excluded from each group respectively. In the unvaccinated group, 340 patients were later discovered to have received the influenza vaccination at immunization camps or a private healthcare institution, and they were added to the vaccinated group. The final analysis was conducted on 3234 influenza vaccinated patient’s vs 5640 unvaccinated ones. (Fig. 1)

## 8. Baseline characteristics

Influenza vaccinated vs unvaccinated, COVID-19 positive patients were substantially older (45 ± 15.2 years vs 37.2 ± 10.5 years, *P* < .001) and primarily Qatari (62.6% vs 32.7%, *P* < .001). The majority of the males (71.1% vs 43%, *P* < .001) were unvaccinated. Although the bulk of the cohort (90%) were nonsmokers, smoking was shown to be considerably higher in the unvaccinated group (10.9% vs 7.8%, *P* < .001). As seen in Table 1, all identified comorbidities were significantly higher (*P* < .001) in the influenza vaccinated group compared to the unvaccinated.

**Table 1:** Baseline characteristics of the study participants.

Baseline variables	Entire cohort n = 8874	Influenza vaccine n = 3234 (36.4%)	No influenza vaccine n = 5640 (63.6%)	*P* value
Age (yr) (mean ± SD)	40.1 ± 12.97	44.95 ± 15.15	37.25 ± 10.547	<.001
Age < 45 yr	6211 (70)	1829 (56.6)	4382 (77.8)	<.001
Age ≥ 45 yr	2658 (30)	1404 (43.4)	1254 (22.2)	
Male, n (%)	6481 (73)	1871 (58)	4610 (82)	<.001
Female, n (%)	2393 (27)	1363 (42)	1030 (18)	
Qatari, n (%)	1123 (13)	703 (22)	420 (7)	<.001
Non-Qatari, n (%)	7751 (87)	2531 (78)	5220 (93)	
Indian subcontinent	5006 (56.4)	1222 (38)	3784 (67)	
Southeast Asia	732 (8.25)	312 (9.6)	420 (7.4)	
Middle east	1801 (20.2)	1079 (33.3)	722 (12.8)	
Africa	1172 (13.2)	542 (16.7)	630 (11.1)	
Europe	65 (0.7)	16 (0.49)	49 (0.86)	
America	35 (0.4)	18 (0.5)	17 (0.3)	
Australia	2 (0.01)	1 (0.03)	1 (0.01)	
Others	61 (0.68)	44 (1.3)	16 (0.2)	
Body mass index (mean ± SD)	29.1 ± 5.80	29.1 ± 5.80	24.2 ± 3.08	.233
Smoker, n (%)	868 (9.8)	252 (7.8)	616 (10.9)	<.001
Diabetes n (%)	1515 (17)	1010 (31)	505 (9)	<.001
Hypertension, n (%)	1441 (16)	906 (28)	535 (9.5)	<.001
Chronic obstructive pulmonary disease, n (%)	30 (0.3)	24 (0.7)	6 (0.1)	<.001
Asthma, n (%)	311 (3.5)	200 (6.2)	111 (2)	<.001
Coronary artery disease/chronic kidney disease/chronic liver disease, n (%)	174 (2)	173 (5.6)	1 (0)	<.001

SD = standard deviation.

## 9. Influenza vaccination and outcomes

The majority of patients, 8464 (95.3%), had mild or asymptomatic COVID-19, whereas 410 (4.6%) had severe or critical COVID-19. The vast majority of Mild (67% vs 61%, P 0.001), Severe (6.3% vs 2.8%, P 0.001), and Critical COVID-19 (0.8% vs 0.4%, *P* < .001) patients were those who had received influenza vaccination. However, in the asymptomatic group, majority of the patients (36% vs 25%, *P* < .001) were unvaccinated. Evaluation of clinical outcomes documented in Table 2 revealed that a total of 1322 (14.9%) required hospitalization of whom 150 (1.7%) required an intensive care admission. All clinical outcomes including hospitalization (22.5% vs 10.5%, *P* < .001), intensive care admissions (3% vs 0.9%, *P* < .001), supportive oxygen therapy (7.4% vs 3.2%, *P* < .001), mechanical ventilation (1.7% vs 0.4%, *P* < .001), death (1.1% vs 0.1%, *P* < .001) and length of stay (18.8 days vs 12 days, *P* .023) were significantly higher in those who had received influenza vaccination.

**Table 2:** Severity of COVID-19 and clinical outcomes.

Outcome variables	Entire cohort n = 8874	Influenza vaccine n = 3234	No influenza vaccine n = 5640	*P* value
Asymptomatic, n (%)	2837 (32)	813 (25)	2024 (36)	<.001
Mild, n (%)	5627 (63.4)	2194 (67.8)	3433 (61)	<.001
Severe, n (%)	361 (4)	202 (6.2)	159 (2.8)	<.001
Critical, n (%)	49 (0.6)	25 (0.8)	24 (0.4)	<.001
Hospitalization, n (%)	1322 (14.9)	728 (22.5)	594 (10.5)	<.001
Intensive care admission, n (%)	147 (1.7)	94 (3)	53 (0.9)	<.001
Oxygen therapy, n (%)	420 (4.7)	238 (7.4)	182 (3.2)	<.001
Mechanical ventilation, n (%)	79 (0.9)	56 (1.7)	23 (0.4)	<.001
Length of stay in ICU median (IQR)	9 (5, 19)	10.5 (5, 20.3)	9 (4.5, 17.5)	.120*
Death, n (%)	42 (0.5)	34 (1.1)	8 (0.1)	<.001

COVID-19 = corona virus disease 2019, ICU = intensive care unit, IQR = inter-quartile-range.

* Mann–Whitney *U* test.

Multivariate logistic regression analysis revealed that patients who were vaccinated had significantly less severe (adjusted odds ratio [OR] 0.685; 95% CI 0.513–0.911, *P* = .010) and critical (adjusted OR 0.345; 95% CI 0.145–0.822, *P* = .016) COVID-19 adjusting potential predictors age, gender and various comorbidities. (Table 3). Vaccinated group also had less asymptomatic COVID-19 (adjusted OR 0.827; 95% CI 0.743–0.919, *P* < .001) and were less likely to require oxygen therapy (adjusted OR 0.696; 95% CI 0.531–0.912, *P* = .009). However, those who were vaccinated had a higher likelihood of mild COVID-19 (adjusted OR 1.281; 95% CI 1.156–1.419, *P* < .001), and hospitalizations (adjusted OR 1.195; 95% CI 1.031–1.384, *P* = .018). Intensive care unit (ICU) admissions (adjusted OR 0.686; 95% CI 0.425–1.11, *P* = .122) and mechanical ventilation (adjusted OR 0.631; 95% CI 0.308–1.295, *P* = .209) were observed to be less in vaccinated group compared to unvaccinated group, however their differences were statistically insignificant (*P* > .05). No significant differences in mortality were noted between vaccinated and unvaccinated group (adjusted OR 1.105; 95% CI 0.348–3.503, *P* = .866). (Table 3 and 4).

**Table 3:** Logistic regression analysis: Severity of COVID -19 with predictor variables.

Outcome variable	Predictor variable	Adjusted odds ratio (OR)	95% confidence interval (CI)	*P* value
Critical COVID-19	Influenza Vaccinated	0.345	0.145–0.822	.016
	Age ≥ 45 yr	2.662	1.098–6.451	.030
	Gender: Male	1.457	0.684–3.103	.329
	Diabetes	1.416	0.661–3.034	.370
	Hypertension	5.609	2.370–13.277	<.001
	Chronic obstructive pulmonary disease	1.484	0.178–12.356	.715
	Asthma	3.077	1.114–8.498	.030
	Coronary artery disease/chronic kidney disease/chronic liver disease	9.119	3.694–22.509	<.001
Severe COVID-19	Influenza vaccinated	0.683	0.513–0.911	.009
	Age ≥ 45 yr	2.356	1.728–3.213	<.001
	Gender: Male	1.117	0.844–1.478	.441
	Diabetes	3.687	2.717–5.003	<.001
	Hypertension	2.064	1.524–2.795	<.001
	Chronic obstructive pulmonary disease	1.917	0.646–5.690	.241
	Asthma	1.278	0.753–2.169	.363
	Coronary artery disease/chronic kidney disease/chronic liver disease	3.173	2.111–4.769	<.001
Mild COVID-19	Influenza vaccinated	1.281	1.156–1.419	<.001
	Age ≥ 45	0.973	0.867–1.091	.637
	Gender: Male	0.673	0.605–0.749	<.001
	Diabetes	1.100	0.948–1.277	.209
	Hypertension	1.118	0.958–1.305	.158
	Chronic obstructive pulmonary disease	0.846	0.361–1.981	.700
	Asthma	1.480	1.122–1.952	.006
	Coronary artery disease/chronic kidney disease/chronic liver disease	0.435	0.314–0.602	<.001
Asymptomatic COVID-19	Influenza vaccinated	0.827	0.743–0.919	<.001
	Age ≥ 45 yr	0.913	0.810–1.030	.141
	Gender: Male	1.523	1.361–1.705	<.001
	Diabetes	0.600	0.507–0.709	<.001
	Hypertension	0.650	0.546–0.773	<.001
	Chronic obstructive pulmonary disease	0.656	0.191–2.258	.504
	Asthma	0.544	0.395–0.749	<.001
	Coronary artery disease/chronic kidney disease/chronic liver disease	0.685	0.427–1.100	.117

COVID-19 = corona virus disease 2019.

**Table 4:** Logistic regression analysis: Clinical outcome variables-death, intensive care admission, hospitalization, mechanical ventilation and oxygen therapy with predictor variables.

Outcome variable	Predictor variable	Adjusted odds ratio (OR)	95% confidence interval (CI)	*P* value
Death	Influenza Vaccinated	1.105	0.348–3.503	.866
	Age ≥ 45 yr	3.191	0.957–10.640	.059
	Gender: Female	0.593	0.240–1.464	.257
	Diabetes	0.916	0.369–2.272	.850
	Hypertension	2.876	0.924–8.950	.068
	Chronic obstructive pulmonary disease	3.400	0.640–18.058	.151
	Asthma	2.142	0.582–7.880	.252
	Coronary artery disease/chronic kidney disease/chronic liver disease	19.735	7.086–54.960	<.001
Intensive Care admission	Influenza vaccinated	0.686	0.425–1.11	.122
	Age ≥ 45 yr	2.435	1.412–4.20	.001
	Gender: Female	1.007	0.657–1.54	.974
	Diabetes	3.072	1.867–5.05	<.001
	Hypertension	3.004	1.810–4.98	<.001
	Chronic obstructive pulmonary disease	1.845	0.482–7.05	.371
	Asthma	1.761	0.864–3.58	.119
	Coronary artery disease/chronic kidney disease/chronic liver disease	5.488	3.260–9.24	<.001
Hospitalization required or not	Influenza vaccinated	1.195	1.031–1.384	.018
	Age ≥ 45 yr	1.798	1.536–2.105	<.001
	Gender: Female	1.182	1.019–1.371	.027
	Diabetes	2.657	2.241–3.151	<.001
	Hypertension	1.927	1.616–2.298	<.001
	Chronic obstructive pulmonary disease	1.959	0.798–4.809	.142
	Asthma	1.627	1.208–2.191	.001
	Coronary artery disease/chronic kidney disease/chronic liver disease	3.112	2.190–4.421	<.001
Mechanical ventilation	Influenza vaccinated	0.631	0.308–1.295	.209
	Age ≥ 45 yr	2.904	1.288–6.548	.010
	Gender: Female	0.689	0.372–1.276	.237
	Diabetes	2.263	1.154–4.440	.017
	Hypertension	3.664	1.743–7.702	.001
	Chronic obstructive pulmonary disease	1.788	0.358–8.937	.479
	Asthma	2.253	0.890–5.701	.086
	Coronary artery disease/chronic kidney disease/chronic liver disease	10.612	5.391–20.88	<.001
Oxygen therapy	Influenza Vaccinated	0.696	0.531–0.912	.009
	Age ≥ 45 yr	2.457	1.837–3.286	<.001
	Gender: Female	0.855	0.655–1.115	.247
	Diabetes	3.107	2.340–4.126	<.001
	Hypertension	2.397	1.804–3.186	<.001
	Chronic obstructive pulmonary disease	2.439	0.896–6.638	.081
	Asthma	1.521	0.946–2.446	.083
	Coronary artery disease/chronic kidney disease/chronic liver disease	4.032	2.752–5.908	<.001

## 10. Discussion

Our study findings show that, patients who received influenza vaccination during the 2019 to 2020 season were less likely to develop severe (32%) or critical (66%) COVID-19. These findings have been replicated in several other studies.^[2–7]^ In a similar study in Qatar, influenza vaccination was found to be 88.9% effective in reducing severe, critical, and fatal COVD-19.^[15]^ However, this mentioned research only included healthcare workers, the majority of whom were younger than 50 years and did not account for comorbidities. This may lead to healthy vaccine bias. Additionally, the nature of the profession of the studied population in this study may influence test seeking behavior; thus, we believe that the results cannot be generalized to the general population. In contrast, our study has been conducted reflecting a real-world cohort, accounting for comorbidities, making it more applicable to the general population. While this does not eliminate healthy vaccine bias, it does reduce its impact. The heterologous effects of vaccines can explain how influenza vaccination may prevent against transmission or reduce the severity of COVID-19. These effects have been noted for almost a century, implying that many vaccines have generalized immune-boosting properties which can cross-protect patients against many pathogens.^[16]^ Heterologous immunity conferred by influenza vaccination, on the other hand, is considered to be transient and short lived.^[17,18]^ We conducted our study over a 9-month period following vaccination, and our cumulative results still show a benefit in severity and hospital outcomes excluding all-cause mortality, implying that influenza vaccination may provide longer cross protection, at least in the context of COVID-19, though exploration of this hypothesis is beyond the scope of our paper. The effect of repeated influenza vaccinations was not investigated in our population. Due to healthy vaccine bias and possible cumulative effect, previous influenza vaccinations before 2019/2020 may have an influence on the incidence of COVID-19 infection, hospitalization, and mortality which was shown by Hosseini-Moghaddam et al.^[19]^ Healthy vaccine bias exists as individuals with health-promoting behaviors are more likely to adhere to the yearly recommended immunization, and regardless of their comorbidities, they are more motivated and engaged in health-promoting and disease-preventing behaviors. However, poor vaccination adherence by those with a deteriorating health status may lead to overestimation of mortality risk reduction.^[20]^ This is particularly true in the case of the influenza vaccine and overestimation of its effectiveness has been shown by Nelson et al^[21]^ and Remschmidt et al^[22]^ in their studies.

In addition to the findings mentioned above, our study found that influenza vaccine recipients had a lower incidence of asymptomatic COVID-19, but a higher incidence of mild COVID-19. The vaccinated group in our study had more comorbidities and a higher mean age, which likely resulted in symptoms and hence more mild COVID-19. Increased reporting of symptoms due to older age and comorbidities may have also led to categorization of these patients as having mild COVID-19 rather than asymptomatic COVID-19. Previous research has however, indicated that greater testing frequency in influenza vaccinated versus unvaccinated individuals may result in an increased incidence of asymptomatic COVID-19 in the vaccinated group.^[21]^ Although we did not look at the testing frequency in our cohort, a higher incidence of asymptomatic COVID-19 in the unvaccinated group mitigates the possibility of increased testing in the vaccinated group. We believe that at the outset of the pandemic, when our study was conducted, everyone was subjected to increased testing owing to both fear of the disease and strict mandatory testing requirements for pandemic control in the state of Qatar.

Previous studies investigating the link between influenza vaccination and the severity of COVID-19 outcomes, including mortality, have yielded mixed results, with the majority indicating decreased mortality and hospital admissions, our study in contradiction reveals higher COVID-19 related hospital admissions and no impact on all-cause mortality among the vaccinated. Increased hospitalization could be explained by an increased health awareness or concern among the vaccinated. Accurately assessing the influence of a vaccination on mortality on the other hand is a tough endeavor, especially when based on observational studies, which can inherently overestimate such benefit. In the case of influenza vaccine, Simonsen and colleagues demonstrate that there are insufficient influenza-related deaths to warrant the conclusion that vaccination can cut total winter mortality among the older population in the United States by up to half.^[23]^ Anderson et al^[24]^ found similar results in a United Kingdom cohort. Furthermore, mortality as an outcome has never been studied in clinical trials. Heterogeneity in immunological response, particularly among older adults, may reconcile effectiveness against COVID-19 related illness with poorer effectiveness against more severe consequences. A recent systematic review by Almadhoon et al^[20]^ also found no significant difference in mortality, hospitalization, or ICU admission. Although our study reports lesser odds of requiring noninvasive oxygen therapy, mechanical ventilation requirement remained unaffected, in contrast to this systematic review that reported a significant reduction in mechanical ventilation.

It can be argued that influenza vaccination’s effect on COVID-19 outcomes was probably significant at a time when COVID-19 vaccination was not available. The innate immunity trained and triggered by influenza vaccination and the effect on protection against other respiratory viral pathogens in the short term may have an impact on severity of COVID -19.^[25,26]^ But with the availability of COVID vaccination almost universally, the question is whether this still remains relevant today? There are multiple unresolved questions regarding the effect of non-COVID-19 vaccinations on COVID-19 illness following the availability of COVID-19 vaccines. The use of the proposed single combined vaccination for influenza and COVID-19 may provide an excellent opportunity to investigate the influence of this combination on COVID-19 as well as influenza-related illness morbidity and mortality.

## 11. Limitations

Our study has limitations given its observational and retrospective nature which can lead to unmeasured confounders and predictors. Other limitations include the inability to identify undiagnosed patients who were not tested for COVID-19 and a degree of healthy vaccine bias.

Additionally, our study lacks consideration of potentially influential factors, such as other vaccines, smoking, and drinking habits, which could impact patient outcomes and introduce confounding variables in the analysis. Furthermore, while ethnicity and socioeconomic conditions may influence the incidence and severity of COVID-19, we were unable to account for these variables in our research.

## 12. Conclusion

Influenza vaccination may significantly reduce the severity of COVID-19 but has no significant effect on all- cause mortality, ICU admissions or mechanical ventilation.

## Author contributions

**Conceptualization:** Merlin Thomas, Shanima Ismail, Mansoor Hameed, Tasleem Raza, Hisham Abdul Sattar.

**Data curation:** Merlin Thomas, Shanima Ismail, Mansoor Hameed, Sabeeha Sayed Tarique Kazi, Theresa Paul, Aasir M Suliman, Sara Saeed Ibrahim Mohamed, Ezzedin A. Salam Ibrahim, Eihab Abd Alla Abd Elrahim Subahi.

**Formal analysis:** Merlin Thomas, Shanima Ismail, Prem Chandra, Theresa Paul, Aasir M Suliman.

**Funding acquisition:** Merlin Thomas, Shanima Ismail.

**Investigation:** Merlin Thomas, Shanima Ismail.

**Methodology:** Merlin Thomas, Shanima Ismail, Prem Chandra, Tasleem Raza.

**Project administration:** Merlin Thomas, Shanima Ismail, Mansoor Hameed, Sabeeha Sayed Tarique Kazi, Theresa Paul, Hisham Abdul Sattar, Aasir M Suliman, Sara Saeed Ibrahim Mohamed.

**Resources:** Aasir M Suliman.

**Software:** Prem Chandra.

**Supervision:** Merlin Thomas, Shanima Ismail, Mansoor Hameed, Tasleem Raza, Hisham Abdul Sattar.

**Validation:** Prem Chandra.

**Visualization:** Shanima Ismail, Theresa Paul.

**Writing – original draft:** Merlin Thomas, Shanima Ismail, Mansoor Hameed, Theresa Paul.

**Writing – review & editing:** Merlin Thomas, Shanima Ismail, Mansoor Hameed, Tasleem Raza.

## References

[1] WHO, 2020. WHO Director-General’s Opening Remarks at the Media Briefing on COVID-19 – 11 March 2020. Geneva, Switzerland: World Health Organization. Available at: https:// www.who.int/dg/speeches/detail/who-director-general-s- opening-remarks-at-the-media-briefing-on-covid-19---11-march- 2020.

[2] Conlon A Ashur C Washer L. Impact of the influenza vaccine on COVID-19 infection rates and severity. Am J Infect Control. 2021;49:694–700. 33631305 10.1016/j.ajic.2021.02.012 PMC7899024

[3] Taghioff SM Slavin BR Holton T. Examining the potential benefits of the influenza vaccine against SARS-CoV-2: a retrospective cohort analysis of 74,754 patients. PloS One. 2021;16:e0255541. 34343191 10.1371/journal.pone.0255541 PMC8330918

[4] Debisarun PA Gössling KL Bulut O. Induction of trained immunity by influenza vaccination – impact on COVID-19. PloS Pathog. 2021;17:e1009928. 34695164 10.1371/journal.ppat.1009928 PMC8568262

[5] Ragni P Marino M Formisano D. Association between exposure to influenza vaccination and COVID-19 diagnosis and outcomes. Vaccines (Basel). 2020;8:675. 33198368 10.3390/vaccines8040675 PMC7711765

[6] Pawlowski C Puranik A Bandi H. Exploratory analysis of immunization records highlights decreased SARS-CoV-2 rates in individuals with recent non-COVID-19 vaccinations. Sci Rep. 2021;11:4741. 33637783 10.1038/s41598-021-83641-y PMC7910541

[7] Candelli M Pignataro G Torelli E. Effect of influenza vaccine on COVID-19 mortality: a retrospective study. Intern Emerg Med. 2021;16:1849–55. 33743150 10.1007/s11739-021-02702-2 PMC7980752

[8] Kalantari S Sadeghzadeh-Bazargan A Ebrahimi S. The effect of influenza vaccine on severity of COVID-19 infection: an original study from Iran. Med J Islam Repub Iran. 2021;35:114. 34956960 10.47176/mjiri.35.114 PMC8683836

[9] Zeng Q Langereis MA van Vliet AL. Structure of coronavirus hemagglutinin-esterase offers insight into corona and influenza virus evolution. Proc Natl Acad Sci U S A. 2008;105:9065–9. 18550812 10.1073/pnas.0800502105 PMC2449365

[10] Wolff GG. Influenza vaccination and respiratory virus interference among department of defense personnel during the 2017-2018 influenza season. Vaccine. 2020;38:350–4. 31607599 10.1016/j.vaccine.2019.10.005 PMC7126676

[11] Yu Y. Herd immunization with childhood vaccination may provide protection against COVID-19. Acta Microbiol Immunol Hung. 2020;67:198–200. 32986604 10.1556/030.2020.01207

[12] Qatar Census December 2020, Available at https://www.psa.gov.qa/en/statistics1/StatisticsSite/Census/census2020.

[13] Ministry of Public health, State of Qatar. Available at https://covid19.moph.gov.qa/EN/Pages/default.aspx.

[14] Wu Z McGoogan JM. Characteristics of and important lessons from the Coronavirus Disease 2019 (COVID-19) Outbreak in China: summary of a report of 72 314 cases from the Chinese center for disease control and prevention. JAMA. 2020;323:1239–42. 32091533 10.1001/jama.2020.2648

[15] Tayar E Abdeen S Abed Alah M. Effectiveness of influenza vaccination against SARS-CoV-2 infection among healthcare workers in Qatar. J Infect Public Health. 2022;16:250–6. 36603377 10.1016/j.jiph.2022.12.016 PMC9791790

[16] Hupert N Marín-Hernández D Gao B. Nixon, heterologous vaccination interventions to reduce pandemic morbidity and mortality: modeling the US winter 2020 COVID-19 wave. Proc Natl Acad Sci USA. 2022;119:e2025448119. 35012976 10.1073/pnas.2025448119 PMC8784160

[17] Lee YJ Lee JY Jang YH. Non-specific effect of vaccines: immediate protection against respiratory syncytial virus infection by a live attenuated influenza vaccine. Front Microbiol. 2018;9:83. 29445364 10.3389/fmicb.2018.00083 PMC5797773

[18] Piedra PA Gaglani MJ Kozinetz CA. Trivalent live attenuated intranasal influenza vaccine administered during the 2003-2004 influenza type A (H3N2) outbreak provided immediate, direct, and indirect protection in children. Pediatrics. 2007;120:e553–64. 17698577 10.1542/peds.2006-2836

[19] Hosseini-Moghaddam SM He S Calzavara A. Association of influenza vaccination with SARS-CoV-2 infection and associated hospitalization and mortality among patients aged 66 years or older. JAMA Netw Open. 2022;5:e2233730. 36169955 10.1001/jamanetworkopen.2022.33730 PMC9520345

[20] Almadhoon HW Hamdallah A Elsayed SM. The effect of influenza vaccine in reducing the severity of clinical outcomes in patients with COVID-19: a systematic review and meta-analysis. Sci Rep. 2022;12:14266. 35995930 10.1038/s41598-022-18618-6 PMC9395333

[21] Nelson JC Jackson ML Weiss NS. New strategies are needed to improve the accuracy of influenza vaccine effectiveness estimates among seniors. J Clin Epidemiol. 2009;62:687–94. 19124221 10.1016/j.jclinepi.2008.06.014

[22] Remschmidt C Wichmann O Harder T. Frequency and impact of confounding by indication and healthy vaccinee bias in observational studies assessing influenza vaccine effectiveness: a systematic review. BMC Infect Dis. 2015;15:429. 26474974 10.1186/s12879-015-1154-y PMC4609091

[23] Simonsen L Reichert TA Viboud C. Impact of influenza vaccination on seasonal mortality in the US elderly population. Arch Intern Med. 2005;165:265–72. 15710788 10.1001/archinte.165.3.265

[24] Anderson ML Dobkin C Gorry D. Effect of influenza vaccination for the elderly on hospitalization and mortality. Ann Intern Med. 2020;173:322–3. 10.7326/L20-0829 32805160

[25] Netea MG Joosten LAB Latz E. Trained immunity: a program of innate immune memory in health and disease. Science. 2016;352:aaf1098. 27102489 10.1126/science.aaf1098 PMC5087274

[26] de Bree LCJ Koeken VACM Joosten LAB. Non-specific effects of vaccines: current evidence and potential implications. Semin Immunol. 39:35–43. 30007489 10.1016/j.smim.2018.06.002

